# Effects of Dietary Energy Levels on Growth Performance, Serum Metabolites, and Meat Quality of Jersey Cattle–Yaks

**DOI:** 10.3390/foods13162527

**Published:** 2024-08-14

**Authors:** Dongqiang Zhang, Min Chu, Qianyun Ge, Ping Yan, Pengjia Bao, Xiaoming Ma, Xian Guo, Chunnian Liang, Xiaoyun Wu

**Affiliations:** 1 Key Laboratory of Animal Genetics and Breeding on Tibetan Plateau, Ministry of Agriculture and Rural Affairs, Key Laboratory of Yak Breeding Engineering, Lanzhou Institute of Husbandry and Pharmaceutical Sciences, Chinese Academy of Agricultural Sciences, Lanzhou 730050, China; zhangdq029@163.com (D.Z.); chumin@caas.cn (M.C.); geqianyun@caas.cn (Q.G.); pingyanlz@163.com (P.Y.); baopengjia@caas.cn (P.B.); maxiaoming@caas.cn (X.M.); guoxian@caas.cn (X.G.); liangchunnian@caas.cn (C.L.); 2 Institute of Western Agriculture, Chinese Academy of Agricultural Sciences, Changji 831100, China; 3 Agricultural Genomics Institute at Shenzhen, Chinese Academy of Agricultural Sciences, Shenzhen 518124, China

**Keywords:** Jersey cattle–yak, metabolizable energy, growth performance, serum parameters, intramuscular fatty acid, meat quality

## Abstract

Energy feed can provide animals with balanced nutrition, thereby enhancing their growth performance. This study aimed to evaluate the effects of dietary energy levels on the growth performance, serum metabolites, and meat quality of Jersey cattle–yaks. A total of 24 male Jersey cattle–yaks were randomly divided into three groups. Each group was fed diets with metabolizable energy levels of 8.21 MJ/kg (LE), 9.50 MJ/kg (ME), and 10.65 MJ/kg (HE), respectively. The HE and ME groups showed significantly higher final body weight, average daily gain (ADG), and feed efficiency compared to the LE group (*p* < 0.05). The glucose (GLU) and total cholesterol (TC) concentrations were significantly increased in the serum of the ME and HE groups (*p* < 0.05). The low-density lipoprotein cholesterol (LDL-C) and alanine aminotransferase (ALT) levels were significantly higher in the serum of the HE group than in the ME group (*p* < 0.05). Blood urea nitrogen (BUN) levels exhibited a significant decrease with increasing metabolizable energy levels in the diet (*p* < 0.05). Increasing dietary energy levels enhances the eye muscle area and intramuscular fat content of Jersey cattle–yaks (*p* < 0.05), with no effect on pH45 min, pH24 h, and shear force. In the HE group, the levels of heneicosanoic acid (C21:0), palmitoleic acid (C16:1), elaidic acid (C18:1n9t), and eicosadienoic acid (C20:2n6) were notably elevated (*p* < 0.05) when compared to the LE group. We concluded that a higher dietary energy level enhanced the growth performance and meat quality traits of male Jersey cattle–yaks.

## 1. Introduction

Yaks are the primary cattle species distributed across the Qinghai–Tibet Plateau and its adjacent alpine and subalpine regions [1]. They provide essential goods for production and living, including meat, milk, wool, fur, leather, labor power, and fuel, serving as the main source of livelihood for local herders [2]. However, due to genetic and environmental constraints, the growth performance of yaks is relatively lower than that of cattle [3]. Hybridizing yaks (*Bos grunniens*) with cattle (*Bos taurus*) for heterosis may increase meat and milk performance [4]. The hybrid of Jersey cattle and yak is known as the Jersey cattle–yak, which exhibits significant heterosis. It can adapt to the hypoxic environment at high altitudes, and is resistant to cold, rough feeding, and has fast growth and development, early sexual maturity, and a high reproductive rate [5]. In the Qinghai–Tibet Plateau and its nearby grazing regions, female Jersey cattle–yaks were raised for both milk production and breeding, whereas their male counterparts were primarily used for meat [6]. Some studies have reported that Jersey cattle–yaks have significantly higher carcass weight, carcass meat yield, and eye muscle area compared to yaks of the same age [7]. Under traditional grazing management, Jersey cattle–yaks were grazed on pastures throughout the year without supplementary feeding [8]. Due to the feeding pattern, Jersey cattle–yaks suffer nutrient deficiencies during the period of dry grass, which directly affects their growth performances [9]. A high-energy diet or feeding regimen can meet maintenance energy needs and optimize the utilization of other nutrients, thereby enhancing growth rates. Therefore, a supplementary feeding regimen is an effective strategy to improve growth performance and shorten the feeding cycles of Jersey cattle–yaks.

Dietary energy is one of the most fundamental nutritional requirements for animals, with nutrient utilization, growth performance, and carcass quality closely tied to the energy level provided by the diet [10]. Increasing the dietary energy level from 5.50 MJ/kg to 6.90 MJ/kg during yak fattening significantly improved feed utilization and daily weight gain [11]. Wang et al. [12] observed that feeding a high metabolizable energy diet (11.68 MJ/kg) increased the dry matter intake, intramuscular fat content, and water-holding capacity of beef in Holstein–Friesian bulls. Kang et al. [13] fed yaks diets with three different net energy levels and found that pre-slaughter live weight, carcass weight, slaughter rate, backfat thickness, and eye muscle area significantly increased in yaks fed high-energy diets (5.32 MJ/kg). Fatty acids influence the flavor, texture, and nutritional quality of meat [14]. Hu et al. [15] found that an adequate net energy level (5.32 MJ/kg) in the diet significantly increased the fatty acid proportion in yak muscle and improved beef quality. Therefore, dietary energy has become a crucial factor in improving the growth traits and meat quality of cattle. Nonetheless, there is a limited understanding of how different dietary energy levels affect the growth performance and meat quality of Jersey cattle–yaks.

In this study, 3-year-old adult male Jersey cattle–yaks were fed diets with different energy levels, and the effects of dietary energy levels on the growth performance, serum metabolites, and meat quality of Jersey cattle–yak were investigated.

## 2. Materials and Methods

### 2.1. Ethics Statement

Protocols for animal experiments were approved by the Animal Administration and Ethics Committee of the Lanzhou Institute of Husbandry and Pharmaceutical Sciences of CAAS (SYXK-2016-0039).

### 2.2. Animals, Experimental Design, and Feeding

Twenty-four male Jersey cattle–yaks aged 3 years with a similar body weight of 320.01 ± 10.25 kg were selected and randomly assigned to three groups of eight individuals each. The diet formulation was designed based on China’s Beef Cattle Feeding Standard. Three diets with different metabolizable energy levels consisted of 40% roughage and 60% concentrates with different energy. The energies of the three concentrates were 8.21 MJ/kg (low-energy group, LE), 9.50 MJ/kg (medium-energy group, ME), and 10.65 MJ/kg (high-energy group, HE), respectively. The roughage consisted of 40% oat hay and 60% whole-plant corn silages. The ingredients and nutritional proportions of the three test diets are presented in Table 1.

The barn was thoroughly cleaned and disinfected before the experiment began, and the Jersey cattle–yaks were assigned numbers and dewormed. The Jersey cattle–yaks were allowed 12 days of acclimatization to their respective diets, facilities, and the barn environment, and a 90 d formal test period. Throughout the trial phase, each Jersey cattle–yak was provided with a total mixed ration (TMR) twice daily at 08:00 and 17:00, with unrestricted access to water and mineralized salt blocks. A daily record was kept of all the feed offered and refused to each steer, and a total daily intake was calculated by subtracting refused feed from all the feed offered.

### 2.3. Feed Nutrient Analysis

Once the formal trial commenced, feed samples were collected weekly and preserved in plastic bags at 4 °C. Feed samples were then dried in an oven at 65 °C until they reached a stable weight, ground, and passed through a 2 mm sieve. The dry matter (DM) content of the feed samples was determined by placing them in an oven at 105 °C for 8 h. Total nitrogen was measured using the Kjeldahl method, and crude protein (CP) was calculated by multiplying the nitrogen content by 6.25 [16]. Neutral detergent fiber (NDF) and acid detergent fiber (ADF) were analyzed using the Ankom method [17].

### 2.4. Growth Performance

During the trial, Jersey cattle–yaks were weighed in the morning before feeding on days 0, 30, 60, and 90. Mean daily gain was calculated as the average of three measurements of individual daily weight gain. The overall weight gain was calculated by subtracting the initial weight from the final weight. The average daily gain (ADG) was determined by dividing the total weight gain by the total number of feeding days during the trial period. To estimate the average dry matter intake (DMI), the difference between the feed provided and the feed left uneaten was recorded for each animal. The feed efficiency for each group of Jersey cattle–yaks was calculated as described by Zhang et al. [18].

### 2.5. Serum Metabolites

Prior to slaughter, blood samples were collected from the jugular vein of Jersey cattle–yaks in each group before their morning feeding. Ten milliliters of blood were collected into vacuum tubes without anticoagulant. The collected samples were stored at a normal temperature for two hours, then centrifuged at 4000 rpm for 15 min at 4 °C to isolate the serum, which was subsequently stored at −20 °C. Serum levels of glucose (GLU), triglycerides (TG), total cholesterol (TC), high-density lipoprotein cholesterol (HDL-C), low-density lipoprotein cholesterol (LDL-C), alanine aminotransferase (ALT), aspartate aminotransferase (AST), and blood urea nitrogen (BUN) were measured using a biochemical automatic analyzer (IDEXX Laboratories, Westbrook, ME, USA).

### 2.6. Meat Quality Traits

Upon the conclusion of the feeding experiment, eight Jersey cattle–yaks from each group were slaughtered. The Jersey cattle–yaks were fasted for 12 h prior to slaughter. Following the slaughter process, the longissimus dorsi (LD) muscle located between the 12th and 13th ribs of the Jersey cattle–yaks was obtained to assess the rib eye area (REA). A Testo 205 pH probe (Testo AG, Schwarzwald, Germany) was used to measure the pH of the LD muscle at 45 min and 24 h after slaughter. The shear force of the LD muscle was measured with a digital meat tenderness meter (C-LM3, Northeast Agricultural University, Harbin, China). The intramuscular fat content of each animal was measured with a 5 g meat sample. Measurements of pH, shear force, and intramuscular fat content were conducted according to the previous and subsequent procedures [19].

### 2.7. Fatty Acid Measurements

The composition of intramuscular fatty acids was analyzed based on a previous study with slight modifications [20]. A 0.50 g sample of Jersey cattle–yak muscle was cut into small pieces and placed into a test tube, and 3 mL of chloroform/methanol (1:1, *v*/*v*) was added to extract the fat. The fat extract was then transferred to a new centrifuge tube, esterified by adding 2 mL of boron trifluoride ethyl ether/methanol (1:3, *v*/*v*) solution, diluted to 1 mL with n-hexane, and filtered through a 0.22 μm organic membrane. Individual and mixed fatty acid methyl ester standard solutions were injected into an Agilent 7890A gas chromatograph (Agilent Technologies, Santa Clara, CA, USA). The relative retention times of the standard solutions were recorded, and the chromatographic peaks were analyzed qualitatively to determine the relative percentages and ratios of saturated fatty acids (SFAs) and unsaturated fatty acids (UFAs) in the LD muscle tissues of Jersey cattle–yaks from different energy groups [21].

### 2.8. Statistical Analysis

The data from this experiment were initially organized and analyzed using Excel 2016. Subsequently, the data were assessed for normality using the Kolmogorov–Smirnov test in SPSS 26.0. The statistical differences in growth performance, serum metabolites, meat quality, and intramuscular fatty acid were analyzed using a one-way ANOVA followed by Duncan’s post hoc testing. A significance level of *p* < 0.05 was considered statistically significant.

## 3. Results

### 3.1. Growth Performance and Apparent Digestibilities

The effects of dietary metabolic energy levels on the body weight, DMI, and feed efficiency of Jersey cattle–yaks are presented in Table 2. Throughout the experiment, Jersey cattle–yaks in the HE and ME groups demonstrated significantly greater final BW, ADG, and feed efficiency than those in the LE group (*p* < 0.05). There were no statistically significant differences in final BW and ADG between the HE and ME groups (*p* > 0.05). The final BW of the LE, ME, and HE groups increased by 41.92%, 45.85%, and 49.73%, respectively, with the ME and HE groups showing significantly higher final BW compared to the LE group (*p* < 0.05). Mean ADG was significantly higher (*p* < 0.05) in the ME and HE groups compared to the LE group, with increases of 13.10% and 22.07%, respectively. A significant downward trend in DMI was observed in the HE and ME groups compared to the LE group (*p* = 0.03).

### 3.2. Serum Biochemical Parameters

The serum metabolites are listed in Table 3. In Jersey cattle–yaks, serum glucose and total cholesterol levels were significantly higher in the HE and ME groups compared to the LE group (*p* < 0.05), while blood urea nitrogen concentrations were significantly lower (*p* < 0.05). Moreover, the HE group showed significantly higher levels of LDL cholesterol and alanine aminotransferase compared to the ME group (*p* < 0.05), while no significant differences were observed between the ME and LE groups (*p* > 0.05). The differences in serum triglycerides, HDL cholesterol, and aspartate aminotransferase activity were not statistically significant (*p* > 0.05) among the three groups.

### 3.3. Meat Quality Characteristics

The effects of dietary energy on meat quality traits are shown in Table 4. Changes in dietary levels had no significant effect on beef pH_45min_ and pH_24h_ (*p* > 0.05). As dietary energy levels increased, there was a decreasing trend in shear force concentration. The HE group exhibited a significantly greater amount of intramuscular fat compared to both the LE and ME groups (*p* < 0.05). Additionally, Jersey cattle–yaks in the ME group had a significantly larger eye muscle area than those in the LE group (*p* < 0.05), with no significant difference compared to the HE group (*p* > 0.05).

### 3.4. Intramuscular Fatty Acid Profiles

The fatty acid composition of Jersey cattle–yak meat across different groups and metabolic energy levels is illustrated in Table 5. The effects of varying dietary energy levels on the concentration of most fatty acids in the LD muscle of Jersey cattle–yaks were not significantly different (*p* > 0.05). As dietary energy levels increased, the heneicosanoic acid (C21:0) content in the LD muscles of the HE group was significantly lower than that in the ME and LE groups (*p* < 0.05). The level of palmitoleic acid (C16:1) in the LD muscles was significantly higher in the HE and ME groups compared to the LE group (*p* < 0.05). The concentration of elaidic acid (C18:1n9t) in the muscles of the ME group was significantly higher than in the LE group (*p* < 0.05) but did not differ significantly from that in the HE group (*p* > 0.05). The eicosadienoic acid (C20:2n6) content in the muscles of the HE group was significantly higher than that in the ME and LE groups (*p* < 0.05). The levels of other saturated, monounsaturated, and polyunsaturated fatty acids did not exhibit significant differences between the groups (*p* > 0.05).

## 4. Discussion

Dietary energy is crucial for animal growth and development, and optimizing energy levels in the diet can maximize animal performance. High-energy diets increase net energy supply and glucose intake, thereby promoting muscle development and fat accumulation [22]. Additionally, higher energy intake up-regulates the expression of genes involved in lipid metabolism and increases intramuscular fat content [11]. Honig et al. [23] demonstrated that feeding high-energy diets can further increase the daily body weights of beef bulls and shorten the fattening cycle. Our research found that the average daily gain of Jersey cattle–yaks in the HE and ME groups increased significantly with higher dietary metabolizable energy, consistent with the results of Li et al. [24]. This suggests that increasing the dietary energy level can effectively increase the ADG of Jersey cattle–yaks, leading to higher feed efficiency. Typically, dry matter intake (DMI) decreases with increasing dietary energy concentration [25]. Ahmad et al. [26] discovered that increased dietary energy intake enhances rumen fermentation efficiency and elevates the concentrations of propionic acid and butyric acid in yaks. Notably, the primary function of propionic acid in the body is to serve as a precursor for glycogen synthesis in the liver [27]. An increase in propionic acid results in higher blood glucose levels, which may, in turn, reduce feed intake [28]. The reduction in dry matter intake observed in this study may be related to this effect.

Fluctuations in dietary nutrient levels lead to alterations in serum metabolites associated with nutrient metabolism, which can act as metabolic signals influencing the growth and development of the animal [29]. Chelikani et al. [30] reported the effects of feeding diets with different nutrient levels on serum metabolites of 3- to 12-month-old dairy heifers. They found that the blood glucose content in serum increased with the increasing gradient of dietary nutrient levels, consistent with our findings. Serum TG are lipid metabolites, and their levels in serum reflect lipid metabolism in animals [31]. Our findings find that TG levels were significantly higher in the ME and HE groups compared to the LE group, suggesting that increased energy intake enhances lipid metabolism in Jersey cattle–yaks. The serum levels of BUN reflect the organism’s metabolism state of protein, and concentrations of BUN have been found to be negatively correlated with nitrogen deposition and the utilization of amino acids [32,33]. It was observed that inadequate energy levels in the diet led to inefficient protein absorption by the body [34]. Our results showed that BUN levels were lower in Jersey cattle–yaks fed high metabolizable energy diets compared to those fed low metabolizable energy diets, suggesting that the Jersey cattle–yaks in the LE group may be consuming insufficient protein. ALT activity increases when animal hepatocytes are stressed or damaged [35]. Serum LDL-C content is an important indicator for evaluating fat metabolism and also reflects the liver’s ability to synthesize proteins [36]. The current findings show that as dietary metabolizable energy levels increased, ALT and LDL-C levels also rose, indicating that higher-energy diets enhance fat metabolism in Jersey cattle–yaks while also imposing additional strain on the liver.

The pH of beef is associated with tenderness and sensory characteristics, indirectly influencing consumer choices [37]. In freshly slaughtered beef, muscle tissue undergoes glycogenolysis, which results in a decrease in pH and an increase in lactic acid production [38]. In this experiment, we found no significant difference in muscle pH among the three groups, indicating that increasing the energy level in diets did not affect the glycolysis in the muscle of Jersey cattle–yaks. Intramuscular fat (IMF) influences meat tenderness by modifying muscle fiber characteristics, and higher IMF content results in a decrease in shear force [39]. We observed a significant increase in intramuscular fat with higher dietary energy levels, while shear force decreased, suggesting that higher energy diets improved tenderness in Jersey cattle–yak beef. The eye muscle area of cattle significantly influences their carcass quality grading. Zhang et al. [40] reported an increase in the loin eye muscle area with higher dietary energy intake. Our research revealed that the HE group had a significantly greater eye muscle area compared to the LE and ME groups. This suggests that higher dietary energy intake can enhance the overall carcass quality of Jersey cattle–yaks. The current study, along with previous studies, confirmed that dietary energy levels improve the carcass quality grading of Jersey cattle–yaks.

Feeding high-concentrate diets increased the proportion of polyunsaturated fatty acids in fattening cattle [41]. The increase in C18:1n9t content can be attributed to feeding grain and high-concentrate-ratio diets to Jersey cattle–yaks. Sun et al. [42] found that C20:2n6 in backfat (BF) and longissimus lumborum (LL) muscles is the fatty acid most closely related to growth and the carcass phenotype. In this experiment, the C20:2n6 content in Jersey cattle–yaks increased significantly in the HE group compared to the ME and LE groups as dietary energy increased, suggesting that increasing dietary energy levels can affect the metabolism of polyunsaturated fatty acids in muscle and revealing an interesting relationship with the growth and carcass characteristics of Jersey cattle–yaks. Wang et al. [43] found that at higher dietary energy and protein levels, there was a gradual decrease in SFA content and a corresponding increase in PUFA and MUFA content in beef. We observed a gradual reduction in the SFA content of Jersey cattle–yaks in the HE group compared to the LE group; however, this change was not statistically significant. This result aligns well with previous studies on SFA trends [44,45]. Fibrous diets with high concentrate ratios pass through the rumen more rapidly, thereby further limiting microbial biohydrogenation [46]. Therefore, dietary energy intake in Jersey cattle–yaks can alter the fatty acid composition and ratio in beef, thereby enhancing its nutritional value and flavor.

## 5. Conclusions

Increasing dietary energy levels enhances growth performance by improving the average daily gain in Jersey cattle–yaks. When the dietary metabolizable energy level reaches 10.65 MJ/kg, blood levels of glucose, total cholesterol, low-density lipoprotein cholesterol, and alanine aminotransferase significantly increase, thereby enhancing fat metabolism in Jersey cattle–yaks. Additionally, only a few fatty acid concentrations, such as heneicosanoic acid, palmitoleic acid, elaidic acid, and eicosadienoic acid, show significant increases in muscle tissue with higher dietary energy levels, potentially improving meat quality to varying extents. This study provides a theoretical foundation for refining the energy requirement standards for Jersey cattle–yaks and establishes a basis for developing fattening technologies under housed feeding and large-scale breeding practices.

## Figures and Tables

**Table 1:** Concentrate supplement composition and nutrient levels.

Item	Diet		
	LE	ME	HE
Ingredients			
Corn	30.00	48.50	61.70
Wheat bran	10.00	7.80	4.00
Soybean oil	-	-	1.00
Spouting corn bran	10.00	10.00	-
Corn white skin	19.50	-	-
Palm kernel cake	8.00	10.00	4.00
Molasses	3.00	3.00	3.00
43 large soybean meal	8.00	8.00	-
43 soybean meal	-	-	10.00
46 cotton meal	6.00	7.00	10.00
MgO	0.30	0.30	0.30
CaHPO_4_	-	0.50	1.00
NaHCO_3_	1.20	1.20	1.00
NaCl	1.00	1.00	1.50
LS	2.00	1.70	1.50
1% ruminant premixed feed	1.00	1.00	1.00
Nutritional ingredient % DM			
Metabolizable energy (MJ/kg)	8.21	9.50	10.65
Crude protein	17.09	16.91	17.00
Neutral detergent fiber	37.39	26.43	17.60
Acid detergent fiber	18.39	13.74	9.24

Note: MgO: magnesium oxide; CaHPO_4_: calcium hydrogen phosphate; NaHCO_3_: sodium bicarbonate; NaCl: sodium chloride; LS: limestone.

**Table 2:** Effects of different energy levels on growth performance of Jersey cattle–yaks.

Item	Groups			SEM	*p*-Value
	LE	ME	HE		
Initial BW (kg)	311.78	329.21	326.92	4.62	0.244
Final BW (kg)	442.50 ^a^	480.14 ^ab^	489.50 ^b^	5.34	0.045
ADG (kg/d)	1.45 ^a^	1.64 ^ab^	1.73 ^b^	0.06	0.041
DMI (kg/d)	11.85 ^a^	11.36 ^ab^	11.26 ^b^	0.05	0.037
Feed efficiency	0.12 ^a^	0.14 ^b^	0.15 ^b^	0.01	0.023

Note: LE: low energy; ME: medium energy; HE: high energy; SEM: standard error of means; BW: body weight; ADG: average daily gain; DMI: dry matter intake. Different letters above the means within a row indicate significant differences (*p* < 0.05).

**Table 3:** Effects of dietary energy levels on serum metabolites of Jersey cattle–yaks.

Item	Groups			SEM	*p*-Value
	LE	ME	HE		
GLU (mmol/L)	3.47 ^a^	3.79 ^b^	3.92 ^b^	0.11	0.043
TG (mmol/L)	0.21	0.23	0.26	0.02	0.063
TC (mmol/L)	2.36 ^a^	2.98 ^b^	3.23 ^b^	0.17	0.034
HDL-C (mmol/L)	1.15	1.21	1.33	0.26	0.182
LDL-C (mmol/L)	0.53 ^a^	0.65 ^ab^	0.76 ^b^	0.02	0.021
ALT (U/L)	18.33 ^a^	24.83 ^ab^	26.35 ^b^	1.22	0.007
AST (U/L)	86.66	83.36	95.58	2.64	0.193
BUN (mmol/L)	4.99 ^a^	4.36 ^b^	4.17 ^b^	0.12	0.026

Note: LE: low energy; ME: medium energy; HE: high energy; SEM: standard error of means; GLU: glucose; TG: triglycerides; TC: total cholesterol; HDL-C: high-density lipoprotein cholesterol; LDL-C: low-density lipoprotein cholesterol; ALT: alanine aminotransferase; AST: aspartate aminotransferase; BUN: blood urea nitrogen. Different letters above the means within a row indicate significant differences (*p* < 0.05).

**Table 4:** Effects of dietary energy levels on meat quality of Jersey cattle–yaks.

Item	Groups			SEM	*p*-Value
	LE	ME	HE		
pH_45min_	6.68	6.55	6.53	0.032	0.637
pH_24h_	5.69	5.66	5.62	0.027	0.938
Shear force (kgf)	17.03	15.95	14.01	0.372	0.513
Intramuscular fat content (g/100 g)	0.75 ^b^	1.26 ^b^	1.91 ^a^	0.021	0.027
Eye muscle area/cm^2^	86.15 ^a^	99.37 ^ab^	108.29 ^a^	8.858	0.019

Note: LE: low energy; ME: medium energy; HE: high energy; SEM: standard error of means. Different letters above the means within a row indicate significant differences (*p* < 0.05).

**Table 5:** Effects of dietary energy levels on fatty acid composition of Jersey cattle–yaks.

Item	Groups			SEM	*p*-Value
	LE	ME	HE		
C12:0	0.14638	0.16838	0.1071	0.0107	0.132
C14:0	2.885	3.95813	2.7667	0.2673	0.133
C15:0	0.27363	0.23675	0.2534	0.0126	0.509
C16:0	25.93738	26.4485	25.5154	0.4278	0.691
C17:0	0.95263	0.86112	0.7829	0.0639	0.575
C18:0	12.76237	12.52863	12.3353	0.1663	0.597
C21:0	0.06225 ^a^	0.14113 ^ab^	0.1554 ^b^	0.0163	0.0001
C14:1	0.40225	0.44425	0.3979	0.0226	0.671
C15:1	0.29137	0.32288	0.3431	0.0103	0.116
C16:1	3.75738 ^b^	4.593 ^ab^	5.5181 ^a^	0.2376	0.003
C17:1	0.54875	0.65725	0.8369	0.0737	0.233
C18:1n9t	2.3775 ^b^	3.53213 ^a^	3.4465 ^a^	0.2012	0.025
C18:1n9c	46.43263	42.66338	44.18738	1.0543	0.356
C20:1	0.4145	0.63988	0.4929	0.0505	0.179
C18:2n6t	0.16025	0.17788	0.2455	0.0161	0.068
C18:2n6c	2.48538	2.44813	2.38098	0.0936	0.907
C18:3n6	0.034	0.10475	0.1384	0.0268	0.278
C20:2n6	0.02 ^c^	0.03162 ^b^	0.047 ^a^	0.003	0.001
C20:4n6	0.05638	0.04288	0.0495	0.0069	0.742
SFA	43.01988	44.342	41.9159	0.6598	0.338
MUFA	54.22413	52.853	55.2226	0.7127	0.413
PUFA	2.756	2.80475	2.8615	0.1054	0.926
PUFA/SFA	0.0639933	0.0629916	0.0703792	0.0023158	0.59
PUFA/MUFA	0.0510274	0.0537507	0.0532992	0.00221773	0.903

Note: LE: low energy; ME: medium energy; HE: high energy; SEM: standard error of means; C12:0: lauric acid; C14:0: myristic acid; C15:0: pentadecanoic acid; C16:0: palmitic acid; C17:0: margaric acid; C18:0: stearic acid; C21:0: heneicosanoic acid; C14:1: myristoleic acid; C15:1: ginkgolic acid; C16:1: palmitoleic acid; C17:1: heptadecenoic acid; C18:1n9t: elaidic acid; C18:1n9c: oleic acid; C20:1: paullinic acid; C18:2n6t: linolelaidic acid; C18:2n6c: linoleic acid; C18:3n6: gamolenic acid; C20:2n6: eicosadienoic acid; C20:4n6: arachidonic acid; SFA: saturated fatty acid; MUFA: monounsaturated fatty acid; PUFA: polyunsaturated fatty acid. Different letters above the means within a row indicate significant differences (*p* < 0.05).

## Data Availability

The original contributions presented in the study are included in the article, and further inquiries can be directed to the corresponding authors.
